# Green Synthesis and Characterization of Silver Nanoparticles Using Rice (*Oryza sativa*) and Spent Coffee (*Coffea robusta*) Grounds from Agricultural Waste^§^

**DOI:** 10.17113/ftb.63.02.25.8925

**Published:** 2025-06

**Authors:** Nithiskanna Nallusamy, Nurul Afifah Mohd Kamal Rufadzil, Jasvini Bala Murally, Jing Zhi Liam, Wan Nor Dalila Wan Fauzi, Hawa Dalily Mohd Jefri, Al-Ashraf Abdullah Amirul, Seeram Ramakrishna, Sevakumaran Vigneswari

**Affiliations:** 1 Institute of Marine Biotechnology, Universiti Malaysia Terengganu, 21030 Kuala Nerus, Terengganu, Malaysia; 2 Faculty of Science and Marine Environment, Universiti Malaysia Terengganu, 21030 Kuala Nerus, Terengganu, Malaysia; 3 School of Biological Science, Universiti Sains Malaysia, 11800 Pulau Pinang, Malaysia; 4 Center for Nanotechnology and Sustainability, National University of Singapore, 119260 Singapore; 5 Ocular Infections and Antimicrobials Research Group, Singapore Eye Research Institute, The Academia, 20 College Road, Discovery Tower, 169856 Singapore

**Keywords:** agricultural waste, green synthesis, rice husks, spent coffee grounds, silver nanoparticles

## Abstract

**Research background:**

Silver nanoparticles (AgNPs) were synthesised using agricultural waste and green synthesis, a sustainable alternative to traditional synthesis techniques that require hazardous chemicals and extensive processing. The AgNPs were produced using spent coffee (*Coffea robusta*) grounds and rice (*Oryza sativa*) husks, both common agricultural wastes rich in bioactive substances such as proteins, flavonoids and phenolic acids that act as natural reducing agents.

**Experimental approach:**

The formation and stability of AgNPs were confirmed using various methods. UV-Vis spectroscopy showed surface plasmon resonance (SPR) peaks at 450 nm, indicating the formation of AgNPs, while Fourier transform infrared spectroscopy (FTIR) identified functional groups responsible for the bio-reduction and stabilisation of the nanoparticles. X-ray diffraction spectroscopy (XRD) confirmed the crystalline, face-centred cubic structure. Zeta potential analysis showed a stable dispersion and particle size analysis showed a consistent size distribution. The antibacterial activity of AgNPs was evaluated by testing their effectiveness against both Gram-positive and Gram-negative bacteria.

**Results and conclusions:**

The AgNPs were synthesised using spent coffee grounds and rice husks, which are rich in biomolecules that serve as effective reducing and stabilising agents. FTIR analysis identified functional groups involved in the reduction and stabilisation of nanoparticles, while XRD confirmed their face-centred cubic (FCC) crystalline structure. Zeta potential measurements showed stable dispersions with particle sizes of AgNPs obtained using spent coffee grounds of approx. 187 nm and of AgNPs obtained using rice husks of 198 nm. The synthesised AgNPs also showed strong antibacterial activity against both Gram-positive and Gram-negative bacteria.

**Novelty and scientific contribution:**

AgNPs were obtained by green synthesis using agricultural waste such as spent coffee grounds and rice husks as natural reducing and stabilising agents. This study highlights the innovative use of biomolecule-rich materials to generate stable AgNPs with strong antibacterial properties and provides a sustainable basis for further development of nanotechnological applications.

## INTRODUCTION

Nanotechnology has become a transformative field dominated by silver nanoparticles (AgNPs) due to their exceptional properties and diverse applications (*1*). Conventional AgNP synthesis often involves hazardous chemicals and complex processes that cause significant environmental concerns (*2*). Therefore, green synthesis has gained attention as a sustainable alternative that uses environmentally friendly materials as reducing agents (*3*). Agricultural waste rich in biomolecules with reducing capabilities offers a promising platform for AgNP production (*4*), which adheres to the principles of sustainable development and circular economy (*5*). Agricultural wastes rich in proteins, phenols and ﬂavonoids act as biological reducing agents in the biological synthesis of AgNPs (*6*) and this approach aids waste management by transforming waste into valuable products. It supports resource conservation by utilising waste materials efficiently, thereby addressing the environmental challenges associated with traditional methods for nanoparticle production (*7*). Although various bio-based approaches have been investigated, the potential of rice husks and spent coffee grounds as precursors for AgNP synthesis remains relatively understudied.

Coffee is a popular non-alcoholic beverage obtained from the roasted seeds of the *Coffea* plant (*8*). Coffee consumption is increasing globally (*9*) and the coffee industry generates significant amounts of waste, including spent coffee grounds (SCG). SCG are a rich source of biologically active compounds, such as flavonoids, phenolic compounds and phytonutrients, but can also contribute to environmental pollution due to high amounts of tannins and caffeine (*10*). The polyphenolic compounds in SCG, including chlorogenic acids and melanoidins, interact with metal salt solutions to form metal atoms through a reduction mechanism (*11*). Additionally, coffee beans contain two main alkaloids, caffeine and trigonelline, and some other compounds such as adenine, xanthine, hypoxanthine and guanine, which can facilitate the formation of nanostructures through oxidative coupling (*12*).

Rice (*Oryza sativa*) husks are a by-product of the rice milling industry often discarded as agricultural waste (*13*). Due to its abundance and renewability, rice husks offer a significant opportunity for sustainable nanoparticle synthesis (*14*). It is a rich source of biologically active compounds, such as phenolic acids and flavonoids, which can facilitate the reduction of metal ions to produce AgNPs (*15*, *16*). The AgNPs synthesised using rice husks are safe for humans and the environment and have been shown to have higher reducing power and the ability to scavenge reactive oxygen species (ROS), which makes them potential antioxidant compounds (*17*). The use of SCG and rice husks as reducing agents provides a cost-effective, sustainable and eco-friendly approach to AgNP production. This method contributes to the sustainable utilisation of agro-waste and a circular bioeconomy, thus helping to mitigate the environmental impact associated with traditional nanoparticle synthesis.

The main focus of this study is to develop a sustainable and environmentally friendly method to synthesise silver nanoparticles (AgNPs) using agricultural waste materials. By utilising rice husks and coffee grounds as reducing agents, we aimed to synthesise nanoparticles and then characterise their properties. This characterisation is essential for evaluating the effectiveness and quality of the produced nanoparticles. Additionally, we investigated the potential benefits of AgNPs in various fields, including antimicrobial, catalytic and biomedical applications. Furthermore, this study paves the way to the advancement of green nanotechnology by demonstrating the feasibility and sustainability of using agricultural wastes for nanoparticle synthesis.

## MATERIALS AND METHODS

### Preparation of spent coffee grounds

Spent coffee ground (SCG) extract was prepared from *Coffea robusta* beans obtained from Ziq Bakery and Cake, Gong Badak, Terengganu, Malaysia. First, the SCG was rinsed with deionized water and filtered to remove excess impurities. The cleaned SCG was then dried in a universal oven (UN55; Memmert, Schwabach, Germany) at 60 °C overnight until complete desiccation, then blended in a blender (MX-GM1011; Panasonic, Shah Alam, Malaysia) to obtain a fine dry powder. A total of 15 g of powdered SCG was mixed with 150 mL deionized water and 30 mL absolute ethanol (8.33 % *m*/*V*) in a 5:1 mass per volume ratio for the extraction of bioactive compounds. This mixture was stirred and heated at 80 °C for 35 min in a water bath (WNB 7; Memmert). After the mixture was cooled down, it was centrifuged at 769×*g* for 15 min using a high-speed floor-top refrigerated centrifuge (CR22N; Hitachi, Hitachinaka, Japan) and then filtered to separate the solid particles from the liquid phase and freeze-dried to preserve the material for further use.

### Preparation of rice husk extract

Rice husks were sourced from MAAS Agros Technology in Kajang, Selangor. A mass of 500 g sample of rice husks was rinsed with deionized water. The rinsed rice husks were dried overnight in a universal oven at 60 °C. The dried rice husk was then powdered to a fine powder in a blender (MX-GM1011; Panasonic).

For the extraction, 300 g of rice husk powder was combined with 1000 mL of deionized water (3:10 *m*/*V* ratio) and heated at 60 °C for 30 min in a water bath. After heating, the mixture was centrifuged at 769×*g* for 15 min (CR22N; Hitachi), followed by filtration and then it was collected and freeze-dried using a freeze dryer (Genesis 35EL Pilot freeze dryer; SP Scientific VirTis, Gardiner, New York, NY, USA) for further use.

### Synthesis of silver nanoparticles using SCG extract

**Fig. S1:**
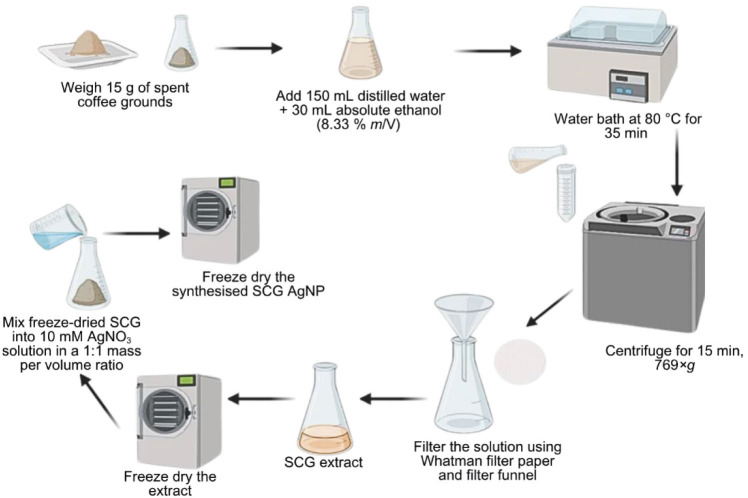
Schematic illustration of the preparation of silver nanoparticles (AgNPs) with addition of spent coffee grounds (SCG) (created using BioRender.com)

The SCG extract was added to 10 mM silver nitrate (Bendosen Laboratory Chemicals, Bendosen, Norway) solution in a 1:1 mass per volume ratio. This mixture was homogenised and stored in a dry, dark place for 5 h (Fig. S1). The change in the colour of the solution indicated the formation of nanoparticles. These changes were observed and recorded hourly from 0 to 5 h.

### Synthesis of silver nanoparticles using rice husk extract

**Fig. S2:**
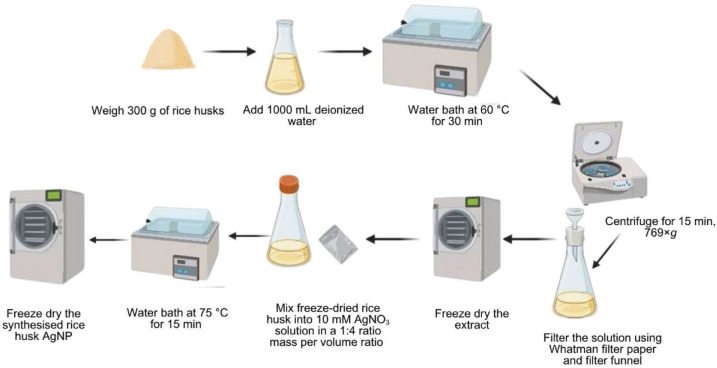
Schematic illustration of the preparation of silver nanoparticles (AgNPs) with the addition of rice husks (created using BioRender.com)

Silver nitrate solution of 10 mM was prepared from solid AgNO_3_. The freeze-dried rice husk extract was mixed with the AgNO_3_ solution in a 1:4 volume ratio (Fig. S2). The reaction mixtures were heated at 75 °C for 15 min in a water bath and then stored in the dark for 96 h to prevent photodegradation. The colour change of the solution indicated nanoparticle formation. The colour changes were observed and recorded hourly.

### UV-Vis spectroscopy

The absorbance of the solution containing nanoparticles was determined using a UV-Vis spectrophotometer (UV-1800, 240 V; Shimadzu, Kyoto, Japan) in a wavelength range from 350 to 650 nm (*18*). The reduction of silver ions obtained by green synthesis was recorded using the UV-Vis spectrum of the reaction of the mixture of silver nanoparticles.

### Structural analysis of AgNPs

The hydrodynamic size of particles (Z average), polydispersity index (PDI) and zeta potential of AgNPs were measured by dynamic light scattering (DLS) (Litesizer 500; Anton Paar, Graz, Austria) (*18*).

The chemical functional groups of synthesised AgNPs were analysed using Fourier transform infrared spectroscopy (FTIR) (IR Traces-100; Shimadzu) in the spectral range of 4000–400 cm^−1^ (*18*).

The profile of freeze-dried AgNPs at 20 and -80 °C was characterised with the X-ray diffraction (XRD) (SmartLab X-ray diffractometer; Rigaku, Tokyo, Japan) using voltage of 40 kV and current of 30 mA (*18*).

### Antimicrobial assay

**Fig. S3:**
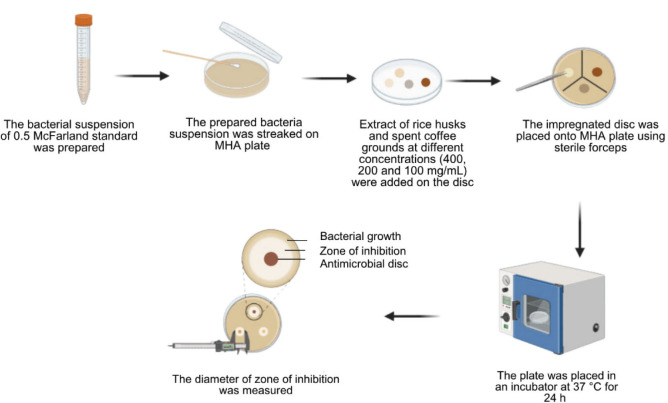
Schematic illustration of the antimicrobial assay of silver nanoparticles (AgNP) produced using rice husks and spent coffee grounds (created using BioRender.com)

The agar disc diffusion method was used to evaluate the antimicrobial activity of silver nanoparticles using rice husk and spent coffee grounds. The synthesised AgNPs showed strong antibacterial properties against Gram-positive bacteria (*Staphylococcus aureus* and *Bacillus subtilis*) and Gram-negative bacteria (*Pseudomonas aeruginosa* and *Escherichia coli*) (Fig. S3). These bacteria were cultured and streaked on the nutrient agar. Using a densitometer, the streaked bacteria were used to prepare the suspension of 0.5 McFarland standard (equivalent to 1.5·10^8^ CFU/mL). The prepared bacterial inoculum was uniformly swabbed on the Mueller-Hinton agar (MHA) plate. The concentrations of 400, 200 and 100 mg/mL of AgNPs, obtained with the addition of rice husk or spent coffee ground extract, were added to the sterile plain disc and placed onto an MHA plate. The zone of inhibition was measured using a vernier calliper after incubating for 24 h at 37 °C. The gentamicin disc was the positive control and distilled water was the negative control, both used to assess the effectiveness of the antimicrobial assay.

### Statistical analysis

All the data are presented as mean value±standard deviation (S.D.). ANOVA and Tukey’s honestly significant difference (HSD) test was used for the statistical analysis with the IBM SPSS Statistics v. 29.0 software, setting the significance at p<0.05 (*19*, *20*).

## RESULTS AND DISCUSSION

### Biosynthesis and spectroscopic analysis of AgNPs derived from agricultural waste

**Fig. S4:**
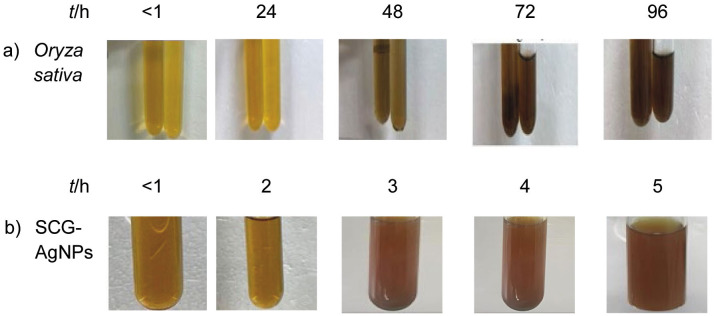
The synthesis of silver nanoparticles (AgNPs) monitored as colour change at different time intervals using: a) rice husk and b) spent coffee ground (SCG)

The synthesis of silver nanoparticles (AgNPs) was successfully achieved through a green approach utilising extracts from *Oryza sativa* (rice husk) and spent coffee grounds as reducing agents. This environmentally friendly method involved the reduction of Ag^+^ ions to AgNPs, visually indicated by a distinct change of colour from light yellow to brownish black (*21*). The colour intensity of rice husk extract in 10 mM AgNO_3_ solution increased from 0 to 96 h (Fig. S4a) of storage and that of spent coffee grounds in 10 mM AgNO_3_ from 0 to 5 h (Fig. S4b). The mechanism, attributed to the excitation of electrons *via* surface plasmon resonance (SPR), confirmed the formation of AgNPs (*22*). Phenolic compounds from the plant extracts contributed a vital role in this bio-reduction process, acting as both reducing and stabilising agents for the nanoparticles (*23*). Optimization of parameters such as metal ion concentration, temperature, and reaction time was essential for achieving optimal AgNP formation (*24*). The interaction of silver ions with phenolic acids from rice husk and coffee grounds was confirmed by the change of colour from light yellow to brownish black, indicating increased stable AgNP formation. Similarly, Vasyliev *et al.* (*25*) found that the black currant pomace extract contained phenolic compounds that may have contributed to the improved stability of AgNPs. The extracts were freeze-dried before being combined with AgNO_3_ for synthesis. Freeze-drying removes 98 % of water from the samples, allowing long-term storage while preserving the bioactive phenolic acids responsible for AgNP formation (*26*).

**Fig. 1:**
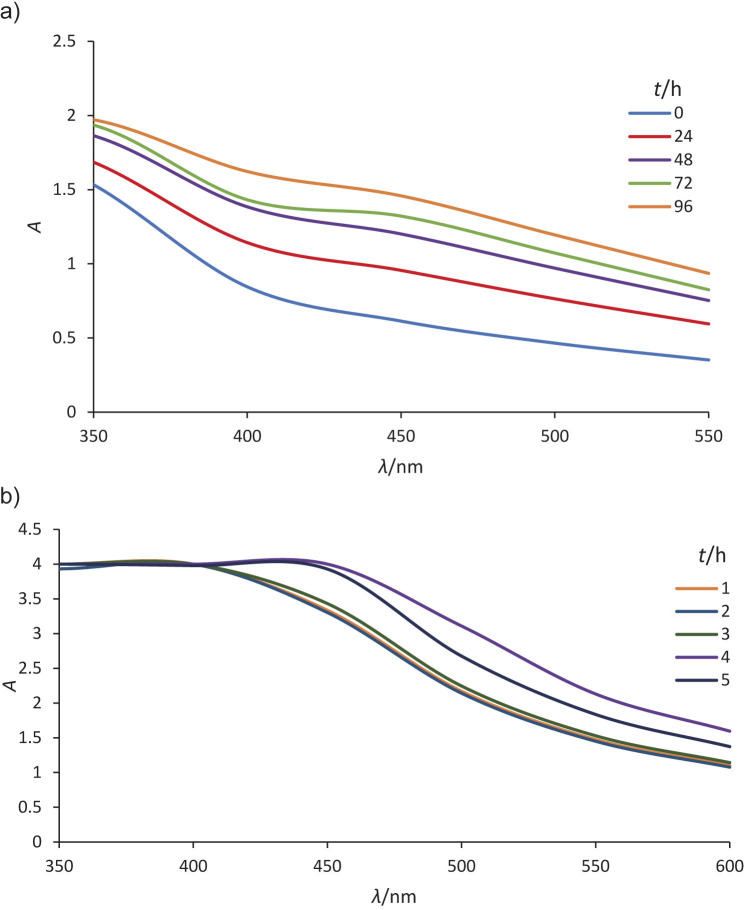
UV-Vis spectra of silver nanoparticles (AgNPs) obtained with the addition of: a) rice husk and b) spent coffee ground (SCG) extracts

The UV-Vis spectra were used to determine the structure of the AgNPs by analysing their free surface electron plasmon oscillations. The SPR peak, indicating AgNP formation, typically appears in the visible range (350-650 nm), which depends on particle size, shape and environment (*27*). Fig. 1a shows that a weak or nearly absent SPR peak at 450 nm was observed for AgNPs obtained from rice husks after 96 h, suggesting the formation of AgNPs. The peaks of phenolic acid components in the spectra obtained for AgNPs were observed at approx. 430 nm, as reported by Liu (*15*).

The concentration of AgNPs is directly proportional to the intensity of the absorption peak, a higher concentration of nanoparticles or larger nanoparticles result in a higher absorption peak (*28*). The increasing absorbance with time suggested the growth of larger particles, indicating a bathochromic shift as the SPR peak shifted towards longer wavelengths (*29*). In the synthesis of AgNP using SCG, a rapid colour change from light to dark brown within 30 min confirmed the formation of AgNPs, with the peak at 450 nm increasing with time and reaching maximum absorbance after 4 h (Fig. 1b). Similarly, silver nanoparticles obtained using spent coffee grounds showed a peak at 450 nm (*30*).

The UV-Vis analysis confirmed AgNP synthesis using both rice husks and SCG extracts, with a consistent SPR peak around 450 nm. These results agree with previous studies that confirm SPR bands in similar ranges (*30*). Spherical metal nanoparticles can only produce a single SPR band, while anisotropic particles may generate two or more SPR bands depending on their shape (*18*). In this study, a single SPR peak was observed, which indicated that the synthesised silver nanoparticles were spherical. This study highlights the feasibility of using sustainable, cost-effective and environmentally friendly methods for AgNP synthesis using agricultural wastes, with potential applications in various fields.

### Analysis of functional groups of AgNP by FTIR

**Fig. 2:**
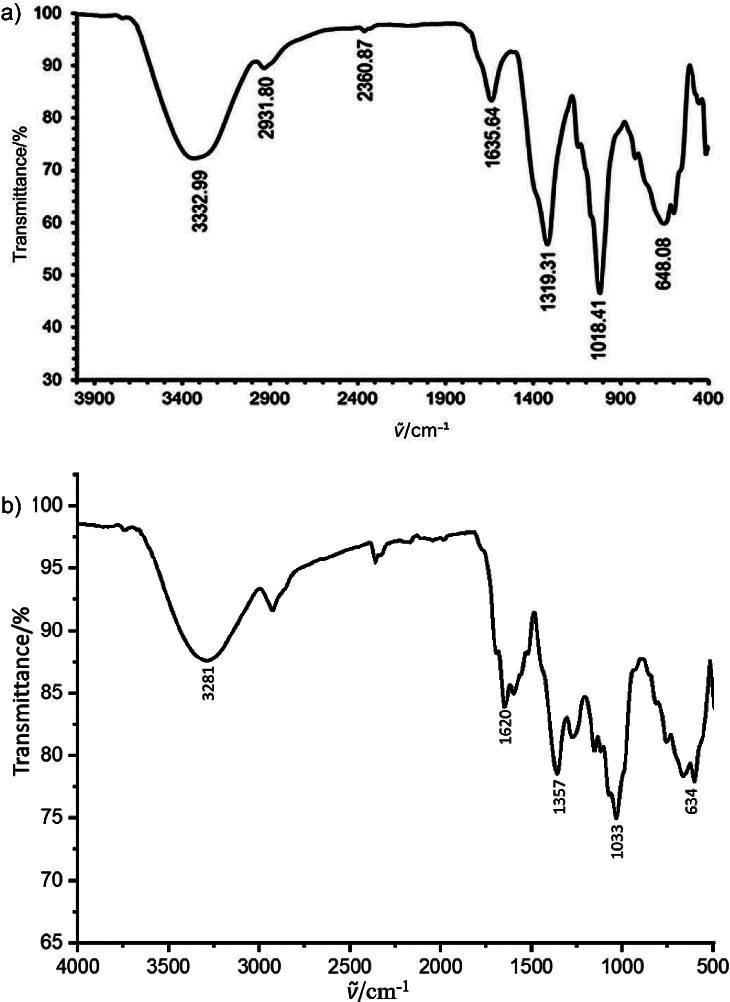
Fourier transform infrared (FTIR) spectra of silver nanoparticles (AgNPs) obtained with the addition of: a) rice husk and b) spent coffee ground extracts

FTIR analysis was carried out to identify the functional groups and biomolecules involved in the bio-reduction of AgNPs derived using rice husks and SCG. This method provides critical insights into the surface chemical composition and reactive sites of the AgNPs that are essential for understanding their surface reactivity (*31*). The FTIR spectra of AgNPs obtained using rice husks in Fig. 2a show several significant absorption peaks. The significant peak at 3332.99 cm^−1^ (O-H stretching) indicates the presence of carboxylic acids (*32*). The O-H stretching probably originates from the phenolic O-H groups in the lignin structure of rice husks (*33*, *34*). Additional prominent peaks were observed at 2931.80 cm^−1^, corresponding to CH_2_ stretching, 2360.87 cm^−1^ (alkynes or ammonium), 1635.64 cm^−1^ (C-N and C-C stretching, associated with proteins or amides), 1319.31 cm^−1^ (N=O stretching, nitro compounds), 1081.41 cm^−1^ (C-N stretching, amines) and 648.08 cm^−1^ (C-Cl stretching, alkyl groups) (*18*, *35*). A broad band at 3286 cm^−1^ and a peak at 2924 cm^−1^ for AgNPs obtained with SCG, as shown in Fig. 2b, indicate different properties (*36*). The peak at 3286 cm^−1^ corresponds to N-H and O-H stretching vibrations and the peak at 2924 cm^−1^ corresponds to asymmetric stretching of the C-H bond in methyl groups (*29*). The broad band at 3286 cm^−1^ suggests the presence of -OH groups in alcohols and phenolic compounds, which function as capping and stabilising agents. For the rice husk-derived AgNPs, the polyphenols and polysaccharides act as capping and stabilising agents (*37*) with the peaks at 1357 and 1273 cm^−1^ associated with the bending vibrations of C-H bonds in methyl and methylene groups, which also affect the size, shape and stability of the AgNPs. The absorption peak at 663.51 cm^−1^ indicated C-Cl stretching vibrations, reflecting the presence of halogen compounds that act as stabilising agents to prevent nanoparticle agglomeration (*38*). Various functional groups were present in rice husk and SCG extracts, as confirmed by FTIR analysis, thus this agricultural waste is a potential resource for the green synthesis of silver nanoparticles, an approach that contributes to the advancement of sustainable and environmentally friendly nanotechnology.

### X-ray diffraction spectroscopy analysis of silver nanoparticles synthesised using rice husk and spent coffee grounds

**Fig. 3:**
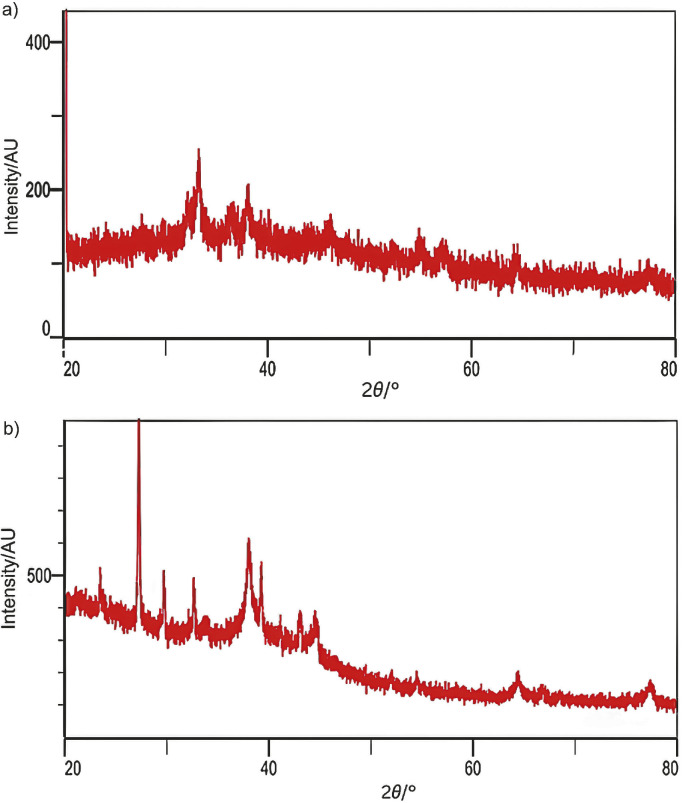
X-ray diffraction (XRD) analysis of silver nanoparticles (AgNPs) obtained with the addition of: a) rice husk and b) spent coffee ground extracts

The molecular and crystalline structures of AgNPs were analysed using X-ray diffraction spectroscopy (XRD), which provides critical insights into the crystalline structure and phase identification of materials. XRD analysis of the molecular and crystalline AgNP structures provided valuable information about the physicochemical properties and degree of crystallinity of the synthesised AgNPs (*39*). The XRD pattern of the AgNPs obtained fusing rice husks (Fig. 3a) showed distinct peaks at 38°, 43°, 64° and 77°, corresponding to (111), (200), (220) and (311) planes, respectively, confirming the presence of a face-centred cubic (FCC) structure (*40*), *i.e.* the synthesised nanoparticles were crystalline. This is consistent with previous studies reporting similar XRD patterns for AgNPs synthesised with apricot and blackcurrant pomace extracts, and rice husks subjected to acid-alkali pretreatment (*15*, *24*). In addition, the XRD analysis of AgNPs obtained from SCG (Fig. 3b) showed prominent peaks in the 2*θ* range from 20° to 80°, with distinct peaks at 27.24°, 38.01° (111), 41.11° (200), 64.3° (220) and 77.3° (311) corresponding to the planes of Bragg's reflection and confirming a face-centred cubic crystalline structure (*38*). Notably, the peak at 38.01° indicates Ag^0^ with an FCC structure and confirms the effective reduction of Ag^+^ ions with both SCG and rice husks (*40*). Overall, these results show the successful formation of crystalline silver nanoparticles using agricultural waste and highlight their potential for sustainable and environmentally friendly applications in nanotechnology (*18*).

### Particle size and zeta potential

**Fig. 4:**
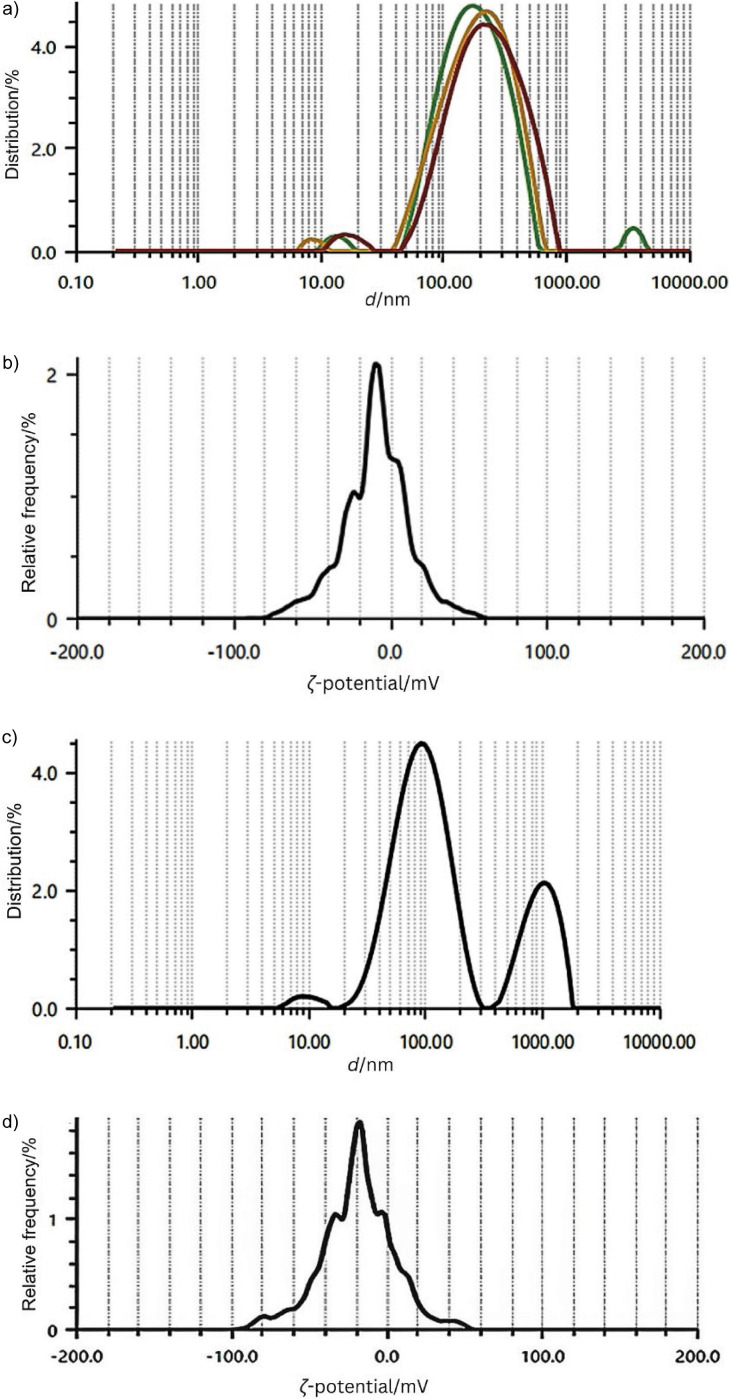
Particle size and zeta potential, respectively, of silver nanoparticles (AgNPs) obtained with the addition of: a) and b) rice husk and c) and d) spent coffee ground extracts

The analysis of AgNPs produced using rice husk and SCG extracts shows that the AgNPs synthesised using rice husks had a larger average size of (198.6±3.6) nm (Fig. 4a), which is attributed to the complex chemical composition of rice husks and affects the formation of nanoparticles. Furthermore, these particles had a zeta potential of (-21.2±2.0) mV and a polydispersity index (PDI) of 26.9 % (Fig. 4b). The AgNPs obtained using SCG had an average size of 187 nm (Fig. 4c), typical for biologically synthesised nanoparticles. However, their stability was moderate, as shown by a zeta potential of -11.8 mV and a PDI of 22.1 % (Fig. 4d), which indicates insufficient electrostatic repulsion and thus the potential for aggregation of particles. The multiple peaks in the particle diameter distribution of rice husks indicate polydispersity and the negative charge of the particles tends to increase stability by inhibiting aggregation (*41*). The negatively charged surface of the nanoparticles contributes to the anionic capping agents, including polyphenols and flavonoids from blackcurrant and apricot pomace extracts, which are coordinated to the surface of the silver nanoparticles (*24*). Optimisation of the synthesis conditions is crucial to obtain AgNPs with the desired size and stability for specific applications. Previous studies have shown that both the concentration of silver nitrate and the pH of the reaction medium significantly influence the properties of the resulting nanoparticles. An increased pH accelerates the reduction of Ag^+^ ions, resulting in smaller AgNPs due to accelerated crystallisation. Additionally, a higher pH can weaken the aggregation of nanoparticles by fully charging the particle surface, and thus increasing electrostatic repulsion. Conversely, a higher AgNO_3_ concentration can also lead to the formation of smaller nanoparticles (*41*).

### Antimicrobial analysis of AgNPs

Organic nanoparticles provide thermal stability, tensile strength, extended shelf life and antibacterial properties (*42*). AgNPs have antibacterial activity against both Gram-positive and Gram-negative bacteria, including multidrug-resistant strains (*39*). Accordingly, this study showed that AgNPs obtained using rice husk and SCG exhibited similar antibacterial activity against both Gram-positive and Gram-negative bacteria (Table 1 and Table 2). Silver nanoparticles obtained from rice husks have antimicrobial activity against bacteria at a concentration of 400 mg/mL. The maximum zone of growth inhibition against *Staphylococcus aureus* and *Bacillus subtilis* is 10 and 12 mm, respectively, as shown in Table 1. The inhibition zone against *Pseudomonas aeruginosa* and *Escherichia coli* is 11 mm for both bacteria at 400 mg/mL. In addition, AgNPs obtained using spent coffee grounds (Table 2) have maximum antimicrobial activity against bacteria at a concentration of 400 mg/mL. The maximum growth inhibition zone against *S. aureus* and *B. subtilis* is 9 and 12 mm, respectively. The inhibition zones against *P. aeruginosa* and *E. coli* are 9 and 11 mm at 400 mg/mL.

**Table 1:** Antibacterial activity of silver nanoparticles (AgNP) obtained with the addition of rice husks against both Gram-positive and Gram-negative bacteria

		*γ*/(mg/mL)					
	Gentamicin	Rice husk AgNP			Rice husk extract	AgNO_3_	Control
Organism		400	200	100	10	1.7	
	*d*(inhibition zone)/mm						
*Pseudomonas aeruginosa*	21.4±0.7	(10.4±0.7)^a^	(9.0±0.7)^ab^	(8.4±0.7)^b^	0	7.0±0.0	0
*Escherichia coli*	20.1±0.2	(11.0±0.0)^a^	(9.0±0.0)^ab^	(8.0±0.0)^b^	0	7.0±0.0	0
*Staphylococcus aureus*	21.0±0.0	(9.4±0.7)^a^	(8.4±0.7)^ab^	(7.4±0.7)^b^	0	7.0±0.0	0
*Bacillus subtilis*	22.0±0.0	(10.4±0.7)^a^	(9.4±0.7)^ab^	(9.4±0.7)^b^	0	7.4±0.7	0

Data are shown as mean value±S.D. (*N*=3). Tukey's honestly significant difference (HSD) test indicates that different letters in superscript denote significant differences within the concentration at p<0.05

**Table 2:** Antibacterial activity of spent coffee ground silver nanoparticles (AgNP) exhibited against both Gram-positive and Gram-negative bacteria

Organism		*γ*/(mg/mL)					
Gentamicin	Spent coffee grounds AgNP			Spent coffee ground extract	AgNO_3_	Control
	400	200	100	10	1.7	
*d*(inhibition zone)/mm						
*Pseudomonas aeruginosa*	21.4±0.7	(9.0±0.0)^a^	(8.0±0.0)^b^	(7.0±0.0)^c^	0	7.0±0.0	0
*Escherichia coli*	20.0±0.0	(10.4±0.7)^a^	(8.0±0.7)^b^	(7.0±0.7)^c^	0	7.0±0.0	0
*Staphylococcus aureus*	22.0±0.0	(9.4± 0.7)^a^	(8.0±0.0)^b^	(7.0± 0.0)^c^	0	7.0±0.0	0
*Bacillus subtilis*	22.0±0.0	(9.4±0.7)^a^	(8.4±0.7)^b^	(7.4± 0.7)^c^	0	7.4±0.7	0

Data are shown as mean value±S.D. (*N*=3). Tukey's honestly significant difference (HSD) test shows that different letters in superscript represent significant differences within the concentration at p<0.05

The antimicrobial efficacy of AgNPs is significantly influenced by their physicochemical properties, including shape, size, concentration and colloidal state (*43*). These properties allow AgNPs to interact with or penetrate cell walls and membranes to exert their antimicrobial effect (*39*). In a recent study, rice husks were found to inhibit the growth of *S. aureus*, *Escherichia coli* and *Salmonella enterica*, demonstrating their potential as an antimicrobial agent (*44*). In addition, rice husk extract was effective against clinical strains of *S. aureus* isolated from skin wound infections (*17*, *45*). It has also been reported that SCG samples were more effective against Gram-positive than Gram-negative bacteria (*46*). The silver nanoparticles synthesised using both rice husks and SCG showed strong antimicrobial properties and their potential for biomedical applications (*47*, *48*).

## CONCLUSIONS

In conclusion, this study successfully demonstrated a green synthesis method for the production of silver nanoparticles (AgNPs) using agricultural waste, namely spent coffee grounds (SCG) and rice husks. The results showed that both rice husk and SCG extracts act as reducing and stabilising agents by utilising their abundance in biomolecules, which includes flavonoids, phenols and other organic components. In the synthesis with both rice husks and SCG, AgNP production was confirmed by the significant colour change during the process and the appearance of surface plasmon resonance peaks at around 450 nm. The potential of these waste materials as bio-reductants was further demonstrated by FTIR analysis, which revealed the precise functional groups involved in the reduction and stabilisation processes. The crystalline face-centred cubic structure of the produced AgNPs, demonstrated by X-ray diffraction studies, is consistent with the known properties of silver nanoparticles. Zeta potential indicated stable nanoparticle dispersions, and particle analysis showed diameters of about 187 nm for AgNPs produced using SCG and 198 nm for AgNPs produced using rice husks. By recycling agricultural waste, this study demonstrates that rice husks and SCG can be used as affordable and sustainable precursors for AgNP synthesis, which is consistent with the ideas of circular economy. The produced AgNPs showed promising antibacterial properties, suggesting potential applications in the environmental and biomedical fields. The production of environmentally friendly, resource-efficient nanoparticles fosters green nanotechnology.
